# Daily GRACE satellite data evaluate short-term hydro-meteorological fluxes from global atmospheric reanalyses

**DOI:** 10.1038/s41598-020-61166-0

**Published:** 2020-03-11

**Authors:** Annette Eicker, Laura Jensen, Viviana Wöhnke, Henryk Dobslaw, Andreas Kvas, Torsten Mayer-Gürr, Robert Dill

**Affiliations:** 1 HafenCity University Hamburg, Hamburg, Germany; 2 German Research Centre for Geosciences (GFZ), Potsdam, Germany; 3 Graz University of Technology, Graz, Austria

**Keywords:** Atmospheric science, Atmospheric dynamics, Hydrology

## Abstract

Changes in terrestrial water storage as observed by the satellite gravity mission GRACE (Gravity Recovery and Climate Experiment) represent a new and completely independent way to constrain the net flux imbalance in atmospheric reanalyses. In this study daily GRACE gravity field changes are used for the first time to investigate high-frequency hydro-meteorological fluxes over the continents. Band-pass filtered water fluxes are derived from GRACE water storage time series by first applying a numerical differentiation filter and subsequent high-pass filtering to isolate fluxes at periods between 5 and 30 days corresponding to typical time-scales of weather system persistence at moderate latitudes. By comparison with the latest atmospheric reanalysis ERA5 of the European Centre for Medium-Range Weather Forecasts (ECWMF) we show that daily GRACE gravity field models contain realistic high-frequency water flux information. Furthermore, GRACE-derived water fluxes can clearly identify improvements realized within ERA5 over its direct predecessor ERA-Interim particularly in equatorial and temperate climate zones. The documented improvements are in good agreement with rain gauge validation, but GRACE also identifies three distinct regions (Sahel Zone, Okavango Catchment, Kimberley Plateau) with a slight degradation of net-fluxes in ERA5 with respect to ERA-Interim, thereby highlighting the potentially added value of non-standard daily GRACE gravity series for hydro-meteorological monitoring purposes.

## Introduction

Global numerical reanalyses of the atmosphere^1–3^, oceans^4^, land surface^5^ and other components of the Earth system are essential tools for climate monitoring and research. Atmospheric reanalyses aim at merging several observational records, while applying physically consistent modeling in an analysis scheme that does not change over time. Recent reanalysis efforts in particular focused on a better representation of atmospheric water fluxes and components of the terrestrial water cycle as specifically relevant quantities for society in terms of water availability for hydroelectricity and human consumption. Validation is typically performed for the individual fluxes utilizing globally distributed observations of precipitation^6^, evapotranspiration^7^, and lateral runoff ^8^. The numerical modeling of clouds and thus atmospheric water fluxes is challenging^9^ but also highly relevant for, e.g., agricultural applications, and has seen rapid progress during the most recent decade with the advent of new satellite observing techniques and its associated data assimilation methodologies^10^.

Satellite gravimetry as realized with the satellite missions GRACE^11^ (2002–2017) and GRACE-FO^12^ (since 2018) has brought fundamentally new insight into mass transport and mass redistribution processes of the Earth system^13^. Gravimetry is the only remote sensing concept that provides quantitative estimates of water mass changes at or beyond the Earth’s surface and has thus contributed unique and highly accurate estimates of ice mass loss from continental ice sheets^14^ and mountain glaciers^15^; seasonal terrestrial water storage changes^16^ and groundwater depletion^17,18^; as well as the contribution of net-inflow of water into the ocean basins to global mean sea-level rise^19^. Time variations in terrestrial water storage as observed by the GRACE mission are closely related to atmospheric net-fluxes of precipitation, evapotranspiration and lateral runoff via the water balance equation^20^ (see Methods section). From monthly GRACE gravity fields, net-fluxes accumulated over 30 days have been compared to flux estimates from different global and regional reanalyses^21–24^ which allowed for the identification of long-term flux biases and even trends^25^.

During recent years, mathematical methods were developed to parameterize global gravity field variations with a temporal sampling of 10 days^26^, 1 week^27^ or even 24 hours^28–33^. Daily sampled gravity fields were successfully applied to study high-frequency wind-driven sea-level changes^34^, short-term transport variations of the Antarctic Circumpolar Current^35^, and the characteristics of major flood events in the Ganges-Brahmaputra catchment^36^.

In view of further progress in daily gravity field modeling from GRACE^37,38^ (see Methods section), we demonstrate in this paper that band-pass filtered atmospheric net-fluxes from satellite gravimetry contain realistic high-frequency signals over the continents and can be used to document quality differences between different atmospheric reanalyses. To this end we first discuss global signals in band-pass filtered GRACE flux data and the latest reanalysis ERA5^39^ of the European Centre for Medium-Range Weather Forecasts (ECWMF) in Section 2, followed by a detailed comparison of the time series for an exemplary grid cell (Section 3). The potential of GRACE to identify improvements realized in ERA5 over its direct predecessor ERA-Interim^40^ is shown in Section 4, particularly with a focus on periods between 5 and 30 days that are exclusively accessible from a new non-standard GRACE gravity series with daily sampling. A comparison with the rather conventional approach of evaluating reanalyses time series against rain gauge observations shows good consistency with the GRACE results but also highlights the potentially added-value of satellite gravimetry for hydro-meteorological monitoring purposes (Section 5).

## Global Patterns of Net-Flux Estimates

**Figure 1:**
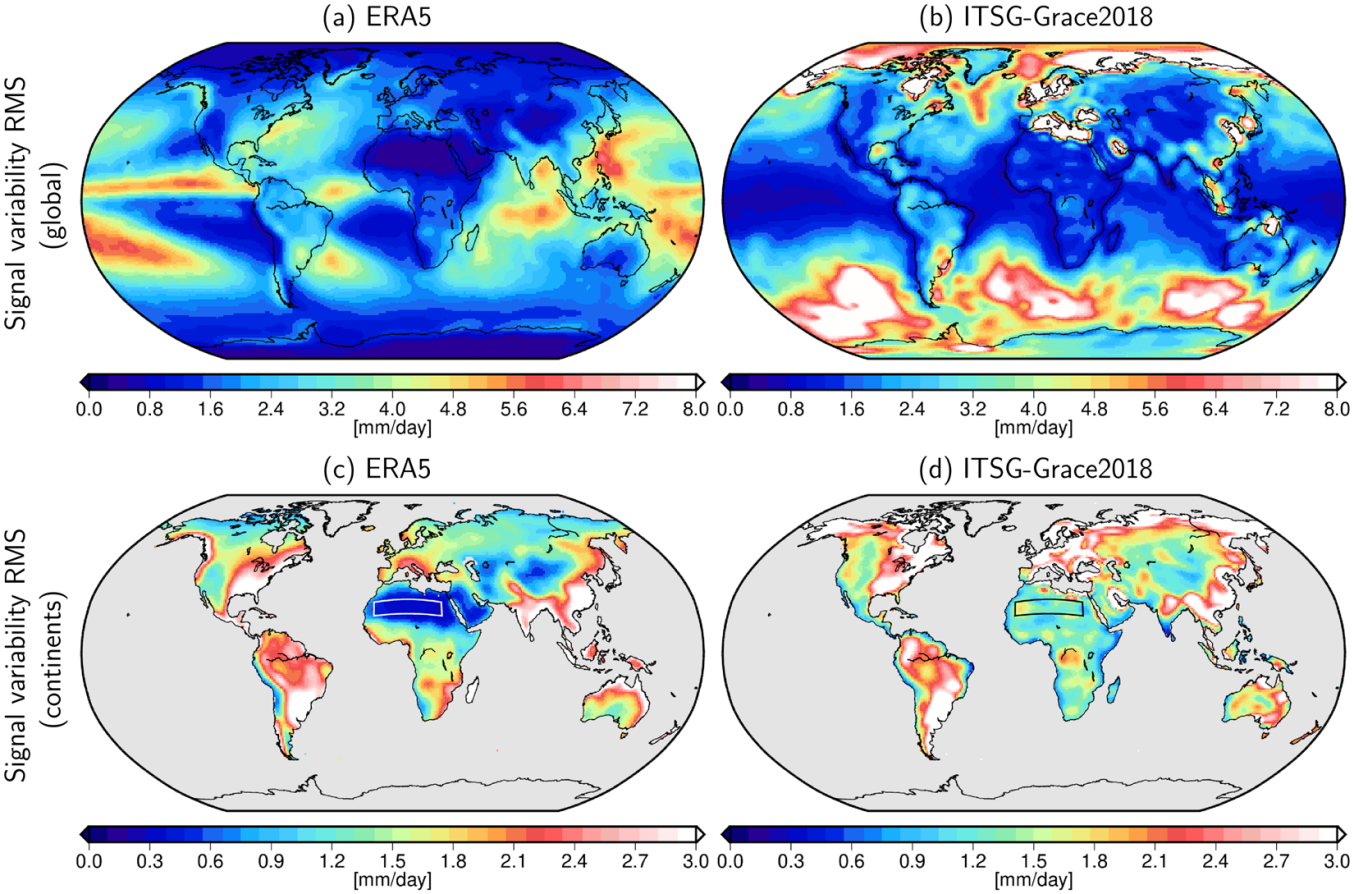
Band-pass filtered signal variability (RMS) of daily ERA5 (left) and daily ITSG-Grace2018 (right) fluxes globally (top row) and on the continents (bottom row). The box over the Sahara indicates the desert region selected for an empirical estimation of the GRACE noise floor.

Although we eventually aim at using GRACE for the evaluation of quality differences between two generations of reanalyses, here we first make a comparison of GRACE and the latest ECMWF reanalysis ERA5 to investigate whether they see comparable high-frequency signals. To this end, band-pass filtered time series of water fluxes were derived from GRACE gravity field models (see Methods Section and Supplementary Information S1) and their root mean squared (RMS) signal variability is displayed together with equally filtered atmospheric net-fluxes from ERA5 in Fig. 1.

We note entirely disparate patterns over the oceans (Fig. 1a,b) related to the fact that short-term fluctuations in sea-level coherent with a mass change are dominated by surface winds instead of atmospheric water fluxes. Time variable gravity observations over the oceans thus provide information about the wind-driven^41^ and regionally also the thermohaline circulation^42^, but are not sensitive to atmospheric water fluxes: precipitation and evaporation mainly affect the density of the near-surface layer of the oceans and any horizontal pressure gradients induced are almost fully compensated with depth in line with the thermal wind equations.

However, over the continents away from the coasts (Fig. 1c,d), we indeed find rather similar features in the fluxes from both ERA5 and GRACE. Estimates from GRACE in arid regions are substantially higher than the values from the reanalysis, reflecting the current level of GRACE observation and analysis noise. For the Sahara desert region (see box outline in Fig. 1), where no substantial day-to-day flux signals can be expected, we find maximum temporal RMS values up to 1.8 mm/day and an area-weighted mean of 1.3 mm/day. Comparative estimates for GRACE releases made available in 2014^43^ and 2016^44^ (both with a mean RMS of 1.7 mm/day in the Sahara desert region) underline the recent progress in understanding GRACE sensor characteristics. This particularly includes a better processing of sensor data from accelerometers and star cameras^45,46^ as well as improved tidal^47^ and non-tidal de-aliasing models^48^, which are commonly regarded as the main error sources in satellite gravimetry from GRACE and GRACE-FO^12^.

We further note that many coastal regions are affected by the limited spatial resolution of GRACE leading to the leakage of signals over the shoreline. The signal RMS in the GRACE time series is considerably larger than in the reanalysis especially in various places along Alaska’s west coast, Hudson Bay, North Sea, Baltic Sea, Black Sea, Persian Gulf, and the Southeast Asian Seas. Similar features can be observed in the Gulf of Carpentaria northwards of Australia. At the same time, GRACE signal variability is dampened with respect to the reanalysis along various coasts in the tropics where no ocean signal is found in the gravity data. We assess the impact of both leakage-in of ocean mass variability onto the continents as well as signal loss of terrestrial water storage variations (see Methods and Supplementary Information S3) and mask all pixels affected by ocean dynamics. The coastal distance of such pixels varies depending on local signal strength and coastal geometry. As a rule of thumb, regions within 200–400 km of the coastline (in extreme cases up to 750 km) are disregarded in the subsequent analysis.

## Atmospheric Net-Fluxes for Aruanã, Brazil

**Figure 2:**
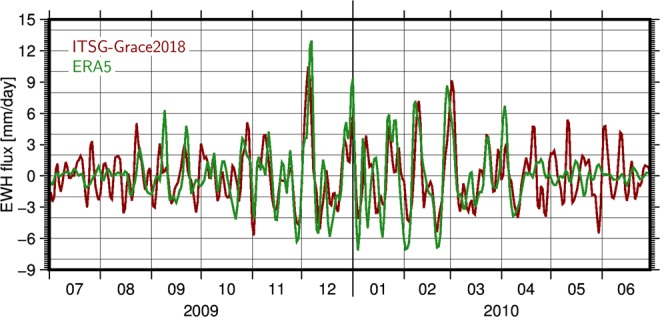
Comparison of band-pass filtered daily flux time series from ITSG-Grace2018 (red) and ERA5 (green) for an exemplary grid cell at Aruanã, Brazil (15°S, 51°W). Fluxes are given as changes in equivalent water height (EWH) in the unit [mm/day].

For the comparison of flux time series from GRACE and reanalyses (here: ERA5) we initially focus on one particular 1° grid cell around the city of Aruanã in the Amazon catchment that is characterized by a humid tropical climate (Fig. 2). Examples for different climatic conditions are given in the Supplementary Information (S4).

We note a generally good agreement between the two data sets, with peaks coinciding especially well in times of large flux variations (November to March) representing the season with highest precipitation rates in the region. The correlation coefficient for the two time series is *ρ* = 0.61 for the 2009/10 time span illustrated here, but shows substantial fluctuations over the year: During the months April until August when precipitation is low, correlation drops to *ρ* = 0.17, but reaches values of up to *ρ* = 0.75 for the wet season during November to March. We also calculate relative explained variances (VAR; see Supplementary Information S2) and note that a substantial part of the GRACE signal can be explained by ERA5 (VAR = 0.33) from November to March, while from April - mid of August the explained variance is negative (VAR = −0.17), which means that subtracting the reanalysis actually does not decrease the signal variability picked up by GRACE. During this time span the signal standard deviation (2.0 mm/day) is only slightly above the GRACE noise floor, indicating that GRACE errors still dominate the signal at times of small fluxes. Investigating the signal characteristics of the band-pass filter applied to both GRACE and ERA5 (see Supplementary Information S1) reveals that periods down to 5 days are detectable in the flux time series. An upper bound of 30 days has been deliberately chosen to demonstrate the added-value of a daily sampled time-series over the conventional monthly GRACE products analyzed previously^25^.

## Evaluation of Global Reanalyses

**Figure 3:**
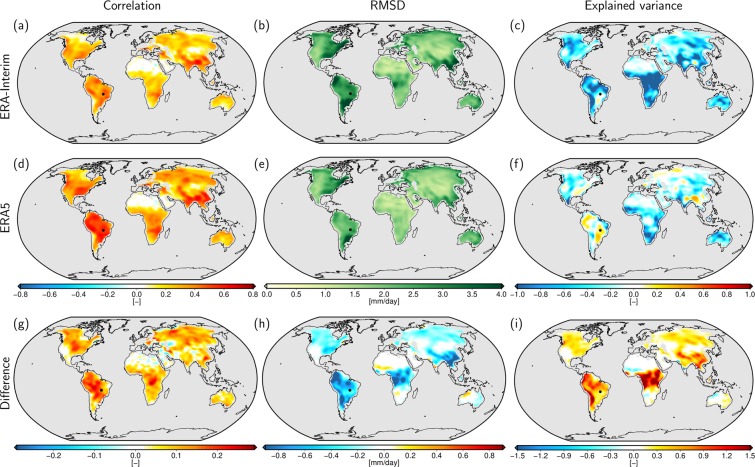
Comparison of three evaluation metrics between the two reanalyses and GRACE: correlation coefficient (left column), root mean squared deviation (middle column), and relative explained variance (right column). The top row shows the respective error measures for ERA-Interim, the middle row for ERA5 and the lower row the difference for each metric (ERA5 minus ERA-Interim). Coastal regions potentially affected by ocean leakage are masked out. The black dot indicates the position of Aruanã, Brazil as discussed in Fig. 2.

We now investigate the potential of GRACE to detect quality differences between subsequently published reanalyses by examining ERA5 together with its predecessor ERA-Interim using data spanning the years 2003–2015. Figure 3 displays three different evaluation metrics calculated for both reanalyses after masking out all regions affected by ocean leakage as explained in the Supplementary Information (S3), where also the definition of the three metrics and a map of the global metrics before masking (Fig. 5 of the Supplement) is given. From the distributions of both correlation coefficients and the root mean squared deviations (RMSD), we note that only very few grid cells, identified by large (dark green) RMSD values, remain that are possibly contaminated by ocean signals, as, e.g., in the Bay of Hanoi, around Beijing, and in the Gulf of St. Lawrence.

We find correlations above 0.3 between GRACE and ERA5 (ERA-Interim) in 55.6% (30.3%) of the land area considered with a maximum value of *ρ* = 0.68 (*ρ* = 0.66) and a median of *ρ* = 0.32 (*ρ* = 0.23). By comparison to the ERA5 signal variability RMS shown in Fig. 1 it becomes obvious that correlations are generally high in regions with large flux signals, such as south-western United States, large parts of South America, the Zambezi area in Africa, or South-East Asia. As expected, hardly any correlations are found in desert areas (Sahara, Atacama, Gobi, and Arabian desert) with very small flux signals. In general, we find higher correlations of GRACE with ERA5 than with ERA-Interim, the difference between both is shown in Fig. 3g. While negative correlation differences especially occur in regions with very small correlation (e.g. Sahara and Arabian Peninsula), GRACE clearly identifies improved correlations for ERA5 in most of the continental areas with particularly large differences in almost the entire North and South American continents, Asia and the central part of Africa.

A more regionally diverse picture is obtained from analyzing the RMSD between GRACE and the reanalyses. Here, particularly ERA-Interim shows large discrepancies to GRACE in South America in the region around the Paraná river basin and in Colombia, in the west of North America and in Southern China. While these areas still show rather large RMSD values also in ERA5, the often negative RMSD differences Fig. 3h indicate improvements in the latest reanalysis compared to its predecessor. Also equatorial Africa exhibits a strong reduction in RMSD. Increasing RMSD values are only observed on the transition between equatorial and arid climate zones in Africa (Sahel and Namibia), in the Okavango catchment, and at the Kimberley Plateau in north-western Australia.

**Figure 4:**
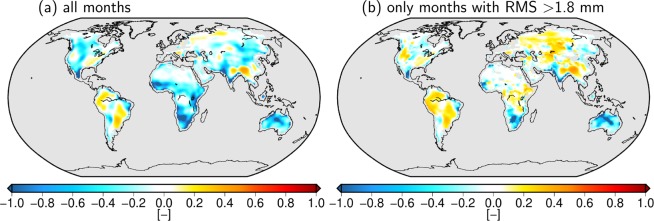
Explained variances (VAR) of the GRACE signal by ERA5 when excluding months with GRACE signal variability below a threshold of 1.8 mm/day. The original result taking into account the complete time series is shown in the left for comparison (same as Fig. 3f).

With respect to the relative explained variance (VAR), we note that the reanalyses only explain a very small part of the GRACE signal variance. In ERA5 some positive areas are visible in Asia and South America with maximum values around 0.45, while for ERA-Interim the explained variance is negative almost everywhere with a maximum value of 0.22. Despite the overall small values, the change of explained variance when moving from ERA-Interim to ERA5 (Fig. 3i) reveals a general improvement of the most recent reanalysis with a quite similar pattern as the one found for the RMSD. In general VAR is the most challenging metric when focusing on high-frequency (high-pass filtered) water flux signals. The very different numbers obtained for individual seasons in the exemplary time series of Aruanã in Fig. 2 (positive VAR in the rainy season and negative VAR in the drier season), however, suggest that the results are strongly influenced by time spans of low GRACE signal variability and thus an unfavorable signal-to-noise ratio (SNR). For a more detailed investigation of this influence, we recomputed the VAR grids for ERA5 excluding time spans with a GRACE signal variability below a certain threshold for each grid cell. We find that the explained variance increases substantially when raising the threshold. From a minimum signal RMS of 1.8 mm/day upwards, which, according to the Sahara test in Section 2 can be regarded as the upper bound of the noise range of GRACE, the explained variances become largely positive with a maximum value of 0.59, see Fig. 4. A more detailed analysis including different choices of thresholds is given in the Supplementary Information S5.

**Figure 5:**
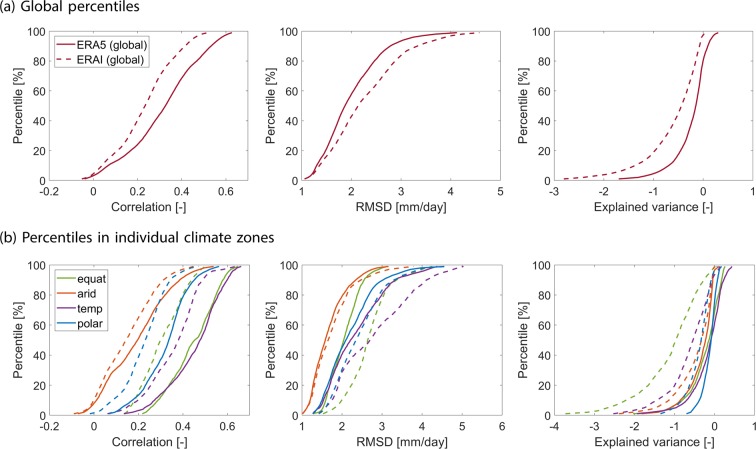
Area-weighted percentiles for correlation (left column), root mean squared deviation (middle column) and explained variance (right column) between GRACE and ERA5 (solid lines) or ERA-Interim (dashed lines). Diagrams in the top row show global percentiles, while in the lower row percentiles for four different climate zones (equatorial, arid, temperate, polar) are displayed.

We also group the statistics calculated for all individual 1° pixels (for the full time series) in terms of area-weighted cumulative distribution functions for each of the three validation metrics (Fig. 5). We note that all global percentiles for correlation, RMSD, and VAR indicate an improvement in ERA5 with respect to ERA-Interim (Fig. 5a). Additionally, the percentiles are shown for individual climate zones^49^ in Fig. 5b with the strongest differences found for all three metrics in the equatorial climate zone.

Here the median correlation, for example, has increased by 50% for ERA5 (*ρ* = 0.45) compared to its predecessor (*ρ* = 0.30), while the median RMSD has decreased by 23% (2.0 mm/day vs. 2.6 mm/day). Considerable improvements are also detected in the temperate climate zone, with an increase of 26% in median correlation and a decrease in 18% of the mean RMSD. In arid climates with much smaller variations in high-frequency atmospheric water fluxes, correlations are generally lower and also the absolute differences between the two reanalyses are less pronounced. However, even in the arid climate zone the relative improvement is clearly detectable with a median correlation increase of 35% from ERA-Interim to ERA5.

## Added Value with Respect to Precipitation Observations

**Figure 6:**
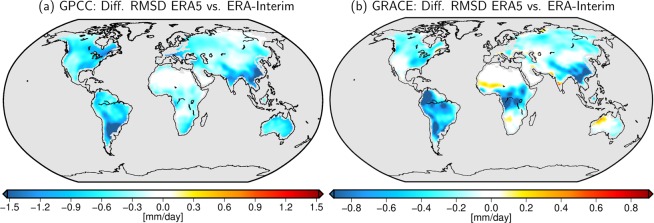
Improvements from ERA-Interim to ERA5 documented by means of smaller RMSDs for precipitation fluxes from reanalyses and GPCC *in situ* observations (left), as well as for net-fluxes of precipitation, evaporation, and lateral runoff from reanalyses and GRACE satellite observations (right; same as Fig. 3h).

We also calculate RMSD changes (ERA5 vs. ERA-Interim) for just precipitation rates against globally gridded rain gauge observations from GPCC^6^ (see Methods) shown in Fig. 6a. Precipitation in reanalyses is not only sensitive to changes in the observing system but also to model physics and the resulting general atmospheric circulation. Therefore, validation against *in situ* measurements is commonly perceived as a critical evaluation measure and a commonly applied diagnostic tool for assessing the quality of atmospheric reanalyses^50^. The overall negative values indicate a smaller RMSD and thus a better agreement of the ERA5 precipitation rates with the rain gauge data when compared to ERA-Interim.

While comparing the regions of particularly large improvements to those detected in the net flux imbalance observed by GRACE (Fig. 6b, same as Fig. 3i), we note a generally good consistency for the validation based on gravity and rain gauges. Strong improvements are found by both data sets in large parts of South America, especially in the south-east around the region of the Paraná River basin. Very high agreement between the two figures is also found in Asia along the Himalaya mountain range and in Southern China. North America sees its largest improvements in the southeastern part of the United States. Hence we conclude that in regions where an improvement in reanalysis *P* can be detected by rain gauge data hinting at precipitation-dominated water fluxes, GRACE is also able to identify a reduction in RMSD with similar magnitudes and geographical distribution.

However, since GRACE observes the net-flux imbalance, it also contains information about the other fluxes *E* and *R* and is, therefore, not redundant to GPCC. As a remote sensing data set, the coverage of satellite gravimetry is globally homogeneous and not depending on the number of hydro-meteorological stations operated (and made available publicly) by a particular country. GRACE thereby documents even more pronounced improvements than GPCC in equatorial Africa, in northern South America at the Colombian Highlands, and the Eastern parts of China. Interestingly, we also note three distinct regions of increasing misfit in the Sahel Zone, the Okavango Catchment, and the Kimberley Plateau, where GRACE-based flux estimates are more aligned to ERA-Interim than ERA5. We speculate that GRACE might point to a deficit in ERA5 in the high-frequency atmospheric fluxes when compared to ERA-Interim that is not detectable from validation against *in situ* rain gauge data alone and advise further studies to elaborate possible reasons for those discrepancies.

## Discussion and Outlook

With a record of about 15.5 years of observations secured by the GRACE mission and with GRACE-FO operating nominally since its launch in May 2018, satellite gravimetry has matured into an operational observing system of global mass change and mass re-distribution. The project team consisting of scientists based in the U.S. and Germany is progressing well towards the low-latency provision of hydrospheric mass change estimates for various applications in the physical Earth sciences. Based on a series of non-standard daily gravity fields, we have demonstrated the potential of GRACE to provide information about atmospheric net-fluxes of water even at short periods between 5 and 30 days. Our approach of relating band-pass filtered water fluxes from GRACE via the terrestrial water balance equation to reanalyses output is generally applicable to all continental regions away from the coasts. We find good correspondence between GRACE flux time series and the most recent state-of-the-art reanalyses ERA5 from ECMWF. The GRACE noise floor is estimated to have an upper bound of around 1.8 mm/day, meaning that in arid climates or during time spans with correspondingly small variations in atmospheric water fluxes, the signal-to-noise ratio limits the reliability of the evaluation.

In regions with short-term flux variations larger than this threshold, our analysis reveals the potential of GRACE to discriminate between atmospheric reanalyses in terms of quality of their atmospheric net-fluxes of water. In the equatorial climate zone, an increase of 50% in median correlation and a decrease of 23% in RMSD was detected in ERA5 with respect to its predecessor ERA-Interim. The assessment of the predictive skill of reanalyses in terms of the explained variance of the GRACE signal is challenging, since in regions and time spans with low signal variability, the GRACE time series is dominated by noise. Even though a clear improvement from ERA-Interim to ERA5 is detected, an evaluation of reanalyses remains difficult because of largely negative values for the explained variance. First experiments show that limiting the evaluation spatially and temporally to regions/time spans with a favorable GRACE signal-to-noise ratio substantially improves the fit. We currently recommend to use GRACE in regions and at times with a minimum signal RMS of 1.8 mm/day, but note that this threshold might change depending on the particular application.

GRACE results largely confirm the improvement in precipitation modeling achieved in ERA5 as already previously known from the comparison against rain gauge observations. In addition, GRACE also identifies degradations of atmospheric net-fluxes of water in ERA5 as compared to ERA-Interim in three distinctive regions not detectable from the rain gauge comparison: the reasons are currently unclear, but should be evaluated further in order to provide potentially valuable feedback to the meteorologic reanalysis community.

Only the recent progress in GRACE data processing has enabled the use of daily GRACE time series for evaluating high-frequency atmospheric fluxes. The accuracy of the previous daily GRACE time series ITSG-Grace2016 would not have been sufficient to carry out such an assessment as demonstrated in the Supplementary Information (S9). It can be assumed that the potential of GRACE data analysis has not yet been fully exploited and that future improvements in gravity field determination can also be expected from the GRACE Follow-On laser ranging instrument measurements. Observations are currently limited to flux variations of periods of 5 days, which might be decreased even further when a constellation of two GRACE-like missions operating simultaneously at differently inclined orbits will be realized in line with multi-disciplinary user requirements^51^. It thus would be sensible to work towards the assimilation of GRACE-based fluxes into numerical weather prediction models in order to fully exploit those ongoingly recorded satellite observations in the field of meteorology.

## Methods

### Daily GRACE gravity fields

In our study we apply the daily solutions of ITSG-Grace2018, which is the latest release of time-variable gravity field models computed at Graz University of Technology^37^. As in the standard processing of monthly GRACE gravity field models, mass variations changing faster than one month are removed prior to the data processing by subtracting the output of geophysical background models from the observations (de-aliasing). These include mass changes caused by ocean, solid Earth and pole tides, as well as non-tidal atmospheric and ocean mass variations^48^. The de-aliasing process thereby removes the gravitational effects of atmospheric mass changes from the daily gravity field models, isolating water storage changes at or below the surface which are caused by vertical and lateral water fluxes in line with the terrestrial water balance equation Eq. (1). For a more detailed discussion on a possible influence of the de-aliasing reductions on the results of this study see the Supplementary Information (S6).

Compared to the standard monthly solutions, the limited satellite ground track coverage during one day does not allow for a stable global gravity field inversion so that additional information has to be introduced. The time series is therefore processed by a Kalman smoother approach similar to the one described by^29^, which introduces in its process model statistical information about the expected time evolution of the gravity field signal. The ITSG-Grace2018 daily solutions apply an auto-regressive (AR) model of order 3 to express the spatio-temporal correlations between epochs, allowing for a better description of the gravity field’s temporal evolution compared to the AR model of order 1 as in previous releases. The process model was derived from the output of the updated Earth System Model of the European Space Agency^52^ (ESA ESM) and primarily includes information about hydrological signal variability, but also residual errors in the background models for atmosphere and ocean dynamics. Since only stochastic information is introduced, no bias towards the ESA ESM is to be expected.

The daily gravity field models are provided as coefficients of a spherical harmonic expansion up to degree *n* = 40 representing water storage anomalies (i.e. deviations from a long-term mean gravity field model) with a spatial resolution of approximately 500 km. No additional spatial filtering is required since spatially correlated noise is effectively suppressed by the Kalman smoother. Since we only focus on the sub-monthly variations, the post-processing steps generally applied to monthly GRACE data (geocenter correction, replacement of coefficient *c*_20_, removal of glacial isostatic adjustment, for example applied in^24^) are omitted. These corrections primarily affect monthly and longer time-scales and would be removed during the high-pass filtering process (see below). We use the time span 2003–2015 for our study, which represents all full years of GRACE sensor data unaffected by the waning battery capacities towards the end of the mission. Even though the Kalman smoother output provides a continuous daily time series without data gaps, all days without GRACE observations were excluded from the analysis. During these time spans the daily solutions are only informed by the process model and thus tend towards a mean trend and annual signal, which vanishes after high-pass filtering. Figure 9 of the Supplementary Information (S7) shows the number of GRACE observations per day, all days with less than 10.000 observations were excluded.

### Atmospheric reanalyses

We use daily gridded precipitation, evapotranspiration, and runoff output fields obtained from two subsequent generations of global atmospheric reanalyses produced by the European Centre for Medium-Range Weather Forecasts (ECMWF). The first and older one is the In-terim Re-Analysis (ERA-Interim)^40,53^. A more recent reanalysis ERA5 is currently being produced by ECMWF within the Copernicus Climate Change Service (C3S)^39^. Important changes relative to ERA-Interim include a higher spatial resolution (31 km compared to 80 km), a strongly improved representation of the troposphere, a better global balance of precipitation and evaporation, improved precipitation over land in the deep tropics, and a new land surface scheme leading to an enhanced consistency of soil moisture and land surface fluxes. Previous studies^54^ found a consistent improvement of land hydrology variables when driving a land surface model by ERA5 vs. ERA-Interim atmospheric forcing. The reanalysis fields were converted from gridded data to a spherical harmonics representation up to degree *n* = 40 in order to obtain a spatial resolution consistent with the GRACE data.

### Daily gridded precipitation data

Daily observational records of precipitation as provided by the Global Precipitation Climatology Centre (GPCC) were applied for additional comparison. We use the most recent version of the GPCC Full Data Daily V.2018^6^, which is based on more than 35,000 gauging stations world-wide and covers the period 1982-2016. The data is available at 1° resolution.

### Band-pass filtered atmospheric fluxes from observed water storage variations

The daily gravity field solutions computed from the GRACE data can be converted to water storage anomalies^55^ and linked to the net flux imbalance of the hydrological fluxes precipitation (*P*), evapotranspiration (*E*), and river discharge (*R*) via the terrestrial water balance equation:

1 $$\frac{dS}{dt}=P-E-R.$$

As a first step, the daily water storage time series *S* is converted to water fluxes (or storage changes) *dS*/*dt* by applying a numerical differentiation filter. Here a simple forward differencing between two subsequent days is not meaningful, as the daily solutions provided by the Kalman smoother are not independent but temporally correlated. The information content of GRACE on daily time scales is closely tied to the ground track patterns of the satellites^29^. Due to the polar orbit configurations, regions around the North and South Pole are effectively sampled every 90 mins, while towards the equator the ground track separation and thus revisit time increases. In a typical, i.e. non-repeat, orbit cycle the globe is fully sampled approximately every 4–5 days.

To account for these sampling characteristics, we use a somewhat broader differentiation filter taking into account the two preceding and the two following days using central weights $-\frac{1}{8},-\frac{1}{4},0,\frac{1}{4},\frac{1}{8}$. We further apply a third-order Butterworth high-pass filter (forwards and backwards to avoid phase shifts) with cut-off frequency of 30 days. Together with the low-pass effect of the 5-point differentiation filter, the result is a band-pass filtered flux time series effectively reducing the analysis to fluxes at periods between 5 and 30 days, which corresponds to the typical time-scale of a mid-latitude cyclone persistence^56^. To allow a meaningful comparison, an equivalent band-pass filter is applied also to the daily reanalyses data on the right-hand side of Eq. (1); for a detailed discussion on the computation steps and the effect of the different filters see Section S1 of the Supplementary Information.

Equation (1) is strictly valid only on the scale of river basins and not on grid-scale, as surface water dynamics in rivers, lakes, and wetlands are not captured in the grid-wise net flux imbalance of the reanalyses. To investigate whether these lateral water transports influence the results of our study, we carried out an additional experiment using a global land surface scheme and discharge model^57^, see Supplementary Information S8. Two different runs of this model were forced by the two atmospheric reanalyses (ERA5 and ERA-Interim) to simulate daily water storage values. Afterward exactly the same analysis steps were applied as to the observed water storage variations from GRACE (i.e. expansion into spherical harmonics up to d/o 40; 5-points differentiation filter to derive fluxes; high-pass filtering). These simulated fluxes are still dominated by ERA5 or ERA-Interim precipitation, but differ from the original reanalyses fluxes *P* − *E* − *R* since they also include all apparent fluxes induced by the simulated surface dynamics. Nevertheless, a comparison with GRACE shows very similar results compared to directly using the reanalyses fluxes as presented above. These simulations thereby confirm that the simplified water balance equation employed has only marginal effects on our results, meaning that surface water variations caused by lateral transport in rivers and lakes do not contribute significantly to the rapid variations in terrestrial water storage observed by GRACE when data averaged over 500 km and at sub-monthly temporal scales is considered. We moreover note that a part of the improvement in the ERA5 net-fluxes over its predecessor indeed appears to be caused by evapotranspiration, which is - in contrast to precipitation - updated in the energy and water balance calculations of the land surface scheme.

### Spatial leakage at the coasts

The low spatial resolution of the daily GRACE data (spherical harmonic degree *n* = 40) does not allow for a strict separation between land and ocean areas along the coast line but results in spatial leakage effects. Additionally, also the Kalman filter approach used for the computation of the gravity field solutions can introduce spurious ocean signal over land. Two different effects need to be considered: Firstly, in areas in which the oceans exhibit larger high-frequency signals than the land areas, the ocean signal leaks onto the continents causing unrealistic signals in continental grid cells. This results in particularly large RMSD values between GRACE and reanalyses at the coast. Secondly, in regions with very small oceanic variability such as the equatorial Atlantic and the Indian Ocean, this leads to a dampening of the GRACE signal on land. We thus mask out all coastal regions that are dominated by coastal leakage as outlined in more detail in the Supplementary Information S5.

## Supplementary information

Supplementary Information.

## References

[1] Harada Y (2016). The JRA-55 Reanalysis: Representation of Atmospheric Circulation and Climate Variability. J. Meteorol. Soc. Jpn. Ser. II.

[2] Saha S (2010). The NCEP climate forecast system reanalysis. Bull. Am. Meteorol. Soc..

[3] Gelaro R (2017). The Modern-Era Retrospective Analysis for Research and Applications, Version 2 (MERRA-2). J. Clim..

[4] Balmaseda MA (2015). The Ocean Reanalyses Intercomparison Project (ORA-IP). J. Oper. Oceanogr..

[5] Reichle RH (2011). Assessment and Enhancement of MERRA Land Surface Hydrology Estimates. J. Clim..

[6] Ziese, M. *et al*. GPCC full data daily version.2018 at 1.0: Daily land-surface precipitation from rain-gauges built on gts-based and historic data, 10.5676/DWD_GPCC/FD_D_V2018_100 (2018).

[7] Jung, M. *et al*. Global patterns of land-atmosphere fluxes of carbon dioxide, latent heat, and sensible heat derived from eddy covariance, satellite, and meteorological observations. *J. Geophys. Res. Biogeosciences* **116**, 10.1029/2010JG001566 (2011).

[8] Ghiggi, G., Humphrey, V., Seneviratne, S. I. & Gudmundsson, L. GRUN: An observations-based global gridded runoff dataset from 1902 to 2014. *Earth Syst. Sci. Data Discuss*. 1–32, 10.5194/essd-2019-32 (2019).

[9] Stephens, G. L. & *et al*. Dreary state of precipitation in global models. *J. Geophys. Res. Atmospheres* **115**, 10.1029/2010JD014532 (2010).

[10] Andersson E (2005). Assimilation and Modeling of the Atmospheric Hydrological Cycle in the ECMWF Forecasting System. Bull. Am. Meteorol. Soc..

[11] Tapley B. D., Bettadpur S., Watkins M., Reigber C. (2004). The gravity recovery and climate experiment: Mission overview and early results. Geophysical Research Letters.

[12] Flechtner F (2016). What Can be Expected from the GRACE-FO Laser Ranging Interferometer for Earth Science Applications?. Surv. Geophys..

[13] Tapley BD (2019). Contributions of GRACE to understanding climate change. Nat. Clim. Chang..

[14] Shepherd A (2012). A Reconciled Estimate of Ice-Sheet Mass Balance. Science.

[15] Gardner AS (2013). A Reconciled Estimate of Glacier Contributions to Sea Level Rise: 2003 to 2009. Science.

[16] Döll P, Fritsche M, Eicker A, Müller Schmied H (2014). Seasonal Water Storage Variations as Impacted by Water Abstractions: Comparing the Output of a Global Hydrological Model with GRACE and GPS Observations. Surv. Geophys..

[17] Rodell M (2018). Emerging trends in global freshwater availability. Nature.

[18] Frappart F, Ramillien G (2018). Monitoring Groundwater Storage Changes Using the Gravity Recovery and Climate Experiment (GRACE) Satellite Mission: A Review. Remote. Sens..

[19] Reager JT (2016). A decade of sea level rise slowed by climate-driven hydrology. Science.

[20] Lorenz C (2014). Large-Scale Runoff from Landmasses: A Global Assessment of the Closure of the Hydrological and Atmospheric Water Balances. J. Hydrometeorol..

[21] Fersch B, Kunstmann H, Bardossy A, Devaraju B, Sneeuw N (2012). Continental-Scale Basin Water Storage Variation from Global and Dynamically Downscaled Atmospheric Water Budgets in Comparison with GRACE-Derived Observations. J. Hydrometeorol..

[22] Riegger J, Tourian MJ, Devaraju B, Sneeuw N (2012). Analysis of GRACE uncertainties by hydrological and hydro-meteorological observations. J. Geodyn..

[23] Springer A, Kusche J, Hartung K, Ohlwein C, Longuevergne L (2014). New Estimates of Variations in Water Flux and Storage over Europe Based on Regional (Re)Analyses and Multisensor Observations. J. Hydrometeorol..

[24] Springer A, Eicker A, Bettge A, Kusche J, Hense A (2017). Evaluation of the Water Cycle in the European COSMO-REA6 Reanalysis Using GRACE. Water.

[25] Eicker A, Forootan E, Springer A, Longuevergne L, Kusche J (2016). Does GRACE see the terrestrial water cycle “intensifying”?. J. Geophys. Res. Atmospheres.

[26] Bruinsma S, Lemoine J-M, Biancale R, Vales N (2010). CNES/GRGS 10-day gravity field models (release 2) and their evaluation. Adv. Space Res..

[27] Dahle, C. *et al*. Gfz grace level-2 processing standards document for level-2 product release 0005: revised edition, january 2013. In *Scientific Technical Report STR12/02–Data, rev. ed*. (Deutsches GeoForschungsZentrum GFZ. Potsdam, 2013).

[28] Kurtenbach, E., Mayer-Gürr, T. & Eicker, A. Deriving daily snapshots of the Earth’s gravity field from GRACE L1b data using Kalman filtering. *Geophys. Res. Lett*. **36**, 10.1029/2009GL039564 (2009).

[29] Kurtenbach E (2012). Improved daily GRACE gravity field solutions using a Kalman smoother. J. Geodyn..

[30] Ramillien GL, Frappart F, Gratton S, Vasseur X (2015). Sequential estimation of surface water mass changes from daily satellite gravimetry data. J. Geod..

[31] Sakumura C, Bettadpur S, Save H, McCullough C (2016). High-frequency terrestrial water storage signal capture via a regularized sliding window mascon product from GRACE. J. Geophys. Res. Solid Earth.

[32] Save, H. & Bettadpur, S. Development of daily “swath” mascon solutions from GRACE. In *EGU General Assembly Conference Abstracts*, vol. 18 (2016).

[33] Gruber C, Gouweleeuw B (2019). Short-latency monitoring of continental, ocean- and atmospheric mass variations using GRACE intersatellite accelerations. Geophys. J. Int..

[34] Bonin Jennifer A., Chambers Don P. (2011). Evaluation of high-frequency oceanographic signal in GRACE data: Implications for de-aliasing. Geophysical Research Letters.

[35] Bergmann I., Dobslaw H. (2012). Short-term transport variability of the Antarctic Circumpolar Current from satellite gravity observations. Journal of Geophysical Research: Oceans.

[36] Gouweleeuw BT (2018). Daily GRACE gravity field solutions track major flood events in the Ganges-Brahmaputra Delta. Hydrol. Earth Syst. Sci..

[37] Mayer-Gürr, T. *et al*. ITSG-Grace2018 - Monthly and Daily Gravity Field Solutions from GRACE, 10.5880/icgem.2018.003, Dataset (2018).

[38] Kvas A (2019). ITSG-Grace2018: Overview and evaluation of a new GRACE-only gravity field time series. J. Geophys. Res. Solid Earth.

[39] Hersbach, H. *et al*. Operational global reanalysis: progress, future directions and synergies with NWP. *ERA Rep. Ser*. (2018).

[40] Dee DP (2011). The ERA-Interim reanalysis: configuration and performance of the data assimilation system. Q. J. Royal Meteorol. Soc..

[41] Hughes, C. W. & Wilson, C. Wind work on the geostrophic ocean circulation: An observational study of the effect of small scales in the wind stress. *J. Geophys. Res. Ocean*. **113**, 10.1029/2007JC004371 (2008).

[42] Landerer FW, Wiese DN, Bentel K, Boening C, Watkins MM (2015). North Atlantic meridional overturning circulation variations from GRACE ocean bottom pressure anomalies. Geophys. Res. Lett..

[43] Mayer-Gürr, T., Zehentner, N., Klinger, B. & Kvas, A. ITSG-Grace2014: A new GRACE gravity field release computed in Graz. In *GRACE Science Team Meeting 2014*, GRACE Science Team Meeting 2014; Conference date: 29-09-2014 Through 01-10-2014 (2014).

[44] Mayer-Gürr, T. *et al*. ITSG-Grace2016 - Monthly and Daily Gravity Field Solutions from GRACE, 10.5880/icgem.2016.007, Dataset (2016).

[45] Bandikova T, Flury J (2014). Improvement of the GRACE star camera data based on the revision of the combination method. Adv. Space Res..

[46] Klinger B, Mayer-Gürr T (2016). The role of accelerometer data calibration within GRACE gravity field recovery: Results from ITSG-Grace2016. Adv. Space Res..

[47] Carrere, L., Lyard, F., Cancet, M. & Guillot, A. Fes 2014, a new tidal model on the global ocean with enhanced accuracy in shallow seas and in the arctic region. In *EGU General Assembly Conference Abstracts*, vol. 17 (2015).

[48] Dobslaw H (2017). A new high-resolution model of non-tidal atmosphere and ocean mass variability for de-aliasing of satellite gravity observations: AOD1b RL06. Geophys. J. Int..

[49] Peel MC, Finlayson BL, McMahon TA (2007). Updated world map of the Köppen-Geiger climate classification. Hydrol. Earth Syst. Sci..

[50] Bosilovich MG, Chen J, Robertson FR, Adler RF (2008). Evaluation of Global Precipitation in Reanalyses. J. Appl. Meteorol. Climatol..

[51] Pail R (2015). Science and User Needs for Observing Global Mass Transport to Understand Global Change and to Benefit Society. Surv. Geophys..

[52] Dobslaw H (2015). The updated ESA Earth System Model for future gravity mission simulation studies. J. Geod..

[53] Berrisford, P. *et al*. The ERA-Interim archive, version 2.0. Report, ECMWF (2011).

[54] Albergel C (2018). ERA-5 and ERA-Interim driven ISBA land surface model simulations: which one performs better?. Hydrol. Earth Syst. Sci..

[55] Wahr J, Molenaar M, Bryan F (1998). Time variability of the Earth’s gravity field: Hydrological and oceanic effects and their possible detection using GRACE. J. Geophys. Res. Solid Earth.

[56] Willett, H. C. & Sanders, F. *Descriptive meteorology* (Academic Press, 1959).

[57] Dill, R. Hydrological model LSDM for operational earth rotation and gravity field variations. In *Scientific Technical Report STR08/09* (Deutsches GeoForschungsZentrum GFZ. Potsdam, 2008).

