# Supplementary material for: Daily GRACE satellite data evaluate short-term hydro-meteorological fluxes from global atmospheric reanalyses
Source: Sci Rep. 2020 Mar 11;10:4504. doi: 10.1038/s41598-020-61166-0 (PMC7066231; doi:10.1038/s41598-020-61166-0)
Supplement: Supplementary file 1 — Supplementary Information. [file 41598_2020_61166_MOESM1_ESM.pdf]

Daily GRACE satellite data evaluate short-term hydro-meteorological fluxes from global atmospheric reanalyses

Annette Eicker[1,*], **Laura Jensen**[1], **Viviana Wöhnke**[1], **Henryk Dobslaw**[2], **Andreas Kvas**[3], **Torsten Mayer-Gürr**[3], **and Robert Dill**[2]

[1]HafenCity University Hamburg, Hamburg, Germany

[2]German Research Centre for Geosciences (GFZ), Potsdam, Germany

[3]Graz University of Technology, Graz, Austria

[*]annette.eicker@hcu-hamburg.de

Supplementary Information

S1: Atmospheric Fluxes from Observed Water Storage Variations

Atmospheric net-fluxes of water are derived from the daily sampled continental water storage estimates observed by the satellite mission GRACE via the terrestrial water balance equation:

$$\frac{dS}{dt} = P - E - R. \tag{1}$$

Here, we discuss in detail the processing of the satellite data for one particular grid cell around Aruanã, Brazil (Fig. 1). The

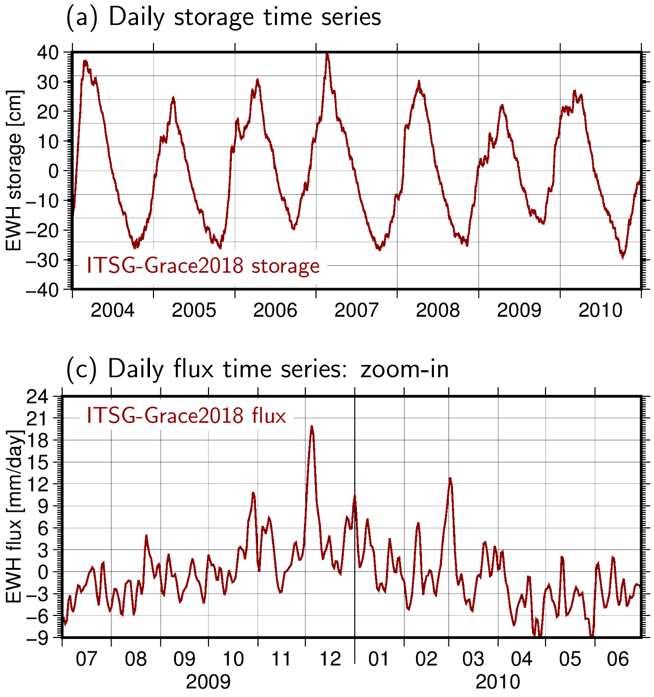

Figure 1. Daily time series for an exemplary grid cell around the city of Aruanã, Brazil (15°S, 51°W): (a) water storages from ITSG-Grace2018 in equivalent water height (EWH), (b) fluxes after applying the differentiation filter, (c) zoom-in on the flux time series for 12 months (July 2009 to June 2010), (d) sub-monthly flux time series after high-pass filtering. For comparison, the un-filtered flux time series is additionally plotted in gray.

initial GRACE water storage time series is shown in Fig. 1a and the resulting flux time series (Fig. 1b) reveals how differentiation increases the high-frequency part of the signal, despite the slight low-pass effect of the applied 5-points differentiation filter (see Methods section of the paper). To allow for a clearer view, additionally a zoom-in on 12 months (July 2009 to June 2010) is presented in Fig. 1c. Since we specifically focus on short-term water storage changes, we apply a Butterworth high-pass filter in forward and backward direction, which conserves the phase. It removes signals with periods longer than 30 days dominating the time series in Fig. 1c to isolate the sub-monthly (band-pass filtered) fluxes (Fig. 1d).

The corresponding ERA5 reanalysis $P-E-R$ time series for the same grid cell is displayed in Fig. 2a with a zoom-in shown in Fig. 2b. To make the reanalyses comparable to the GRACE-derived fluxes, an equivalent band-pass filter was designed: In a first step, the low-pass effect of the 5-points GRACE differentiation filter was mimicked by a central phase preserving filter with weights $\frac{1}{22}, \frac{1}{4}, \frac{9}{22}, \frac{1}{4}, \frac{1}{22}$. Comparing the gray line in Fig. 2c to the original fluxes (green line) shows the smoothing effect. The suitability of this filter was verified in a synthetic experiment by first integrating the ERA5 flux series in time to derive water storages and subsequently using the differentiation filter to re-build GRACE-like water fluxes (orange line in Fig. 2c). The good agreement between the gray and orange lines confirms the equivalence of the two low-pass filters, see also the small inlay in Fig. 2c. Subsequently, the Butterworth high-pass filter was applied to the reanalysis time series to compute band-pass filtered water fluxes corresponding to those derived from GRACE (green line in Fig. 2d).

The effect of the different filters on the signal was investigated as follows: Fig. 3a shows the theoretical amplitude response of the low-pass filter (green), the high-pass filter (blue), and the resulting band-pass filter (red). The figure reveals that the 50%

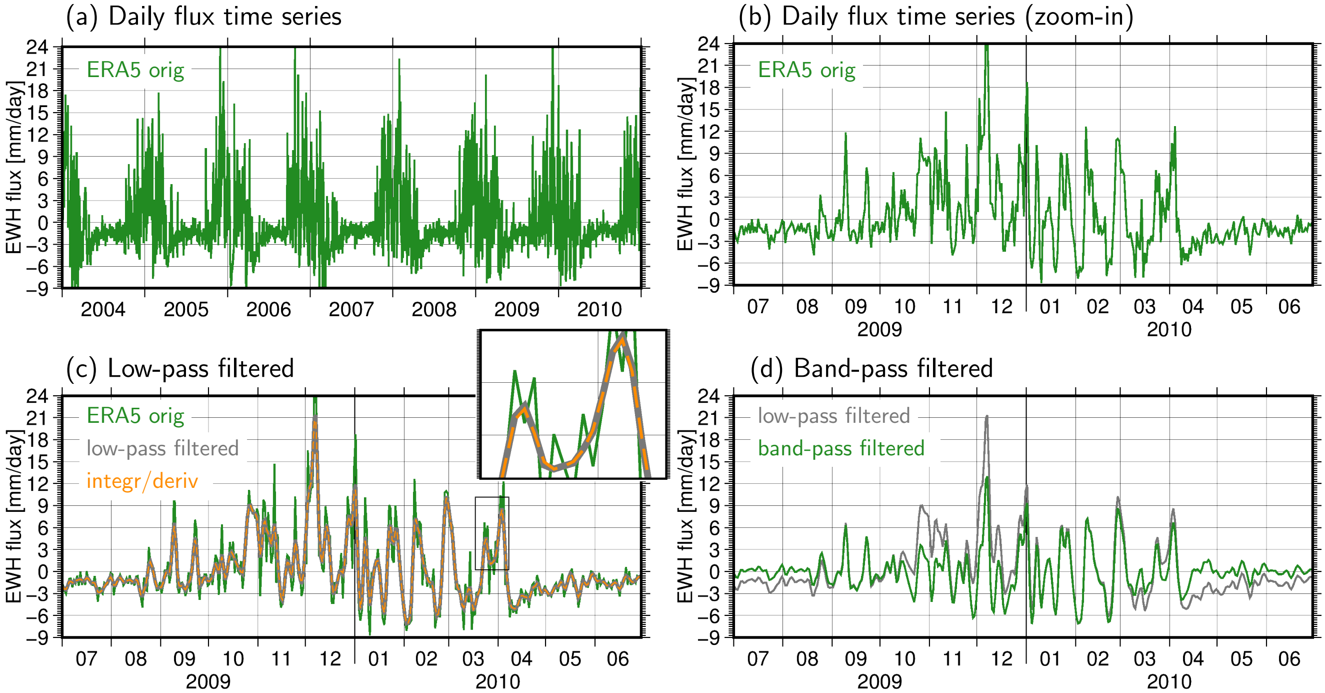

Figure 2. Daily ERA5 time series of atmospheric net-fluxes for an exemplary grid cell around the city of Aruanã, Brazil (15°S, 51°W): (a) fluxes $P-E-R$ from ERA5, (b) zoom-in on the flux time series for 12 months (July 2009 to June 2010), (c) time series after applying a 5-points low-pass filter to the original fluxes (gray) and after integration from flux to storage and subsequent application of the GRACE-like differentiation filter (orange). The inlay shows a zoom-in on the black box for March and April 2010 to better display the small difference between gray and orange line. (d) band-pass filtered flux time series after high-pass filtering (green line). The low-pass filtered time series (gray) is additionally plotted for comparison.

amplitude ratio of the filtered and unfiltered time series is reached at 30 and 5 days, representing the upper and lower boundaries of the frequencies to be expected in the band-pass filtered GRACE or ERA5 time series. The influence of the filters on the time series can be confirmed by examining an exemplary amplitude spectral density, shown in Fig. 3b for the grid cell around Aruanã, Brazil. The black line represents the original (unfiltered) ERA5 signal, the green and red line stand for the band-pass filtered fluxes of ERA5 and GRACE, respectively. A moving average (51 points) has been applied to smooth the rather noisy spectra before display for a clearer illustration. The effect of the high-pass filter is clearly visible as it diminishes periods longer than 30 days. In the higher frequencies, the low-pass filter dampens the filtered fluxes in correspondence with the amplitude response (the ratio between the red and black line in Fig. 3b equals the red line in Fig. 3a). Furthermore, the amplitudes in the GRACE signal correspond very well to the amplitudes in the band-pass filtered ERA5 spectrum. Thus it can be concluded that the Kalman filter processing of the daily GRACE solutions does not introduce any additional temporal smoothing and that the 5-30 days band-pass filter is a good representation of the actual signal content to be expected from the GRACE data.

S2: Evaluation Metrics for Fluxes

The agreement between the GRACE and reanalyses time series was quantified by three different statistical measures that were computed globally for each continental grid cell: the correlation coefficient (ρ), the root mean squared deviation ($RMSD$), and the explained signal variance (VAR). In the equations below x_i always denotes the model (atmospheric reanalyses) and y_i describes the observations (daily GRACE fluxes) serving as the reference here.

The Pearson product-moment correlation coefficient ρ explains the strength and direction of a linear relationship between the GRACE and ERA5-derived flux estimates and can be computed according to

$$\rho = \frac{\sum\left[(x_i-\bar{x})(y_i-\bar{y})\right]}{\sqrt{\sum(x_i-\bar{x})^2}\cdot\sqrt{\sum(y_i-\bar{y})^2}} \tag{2}$$

with $\bar{x}$ and $\bar{y}$ denoting the mean value of the GRACE and reanalysis time series, respectively. Due to the applied high-pass filtering, both mean values result in $\bar{x}=\bar{y}=0$. Generally, the correlation coefficient is well suited for indicating linear

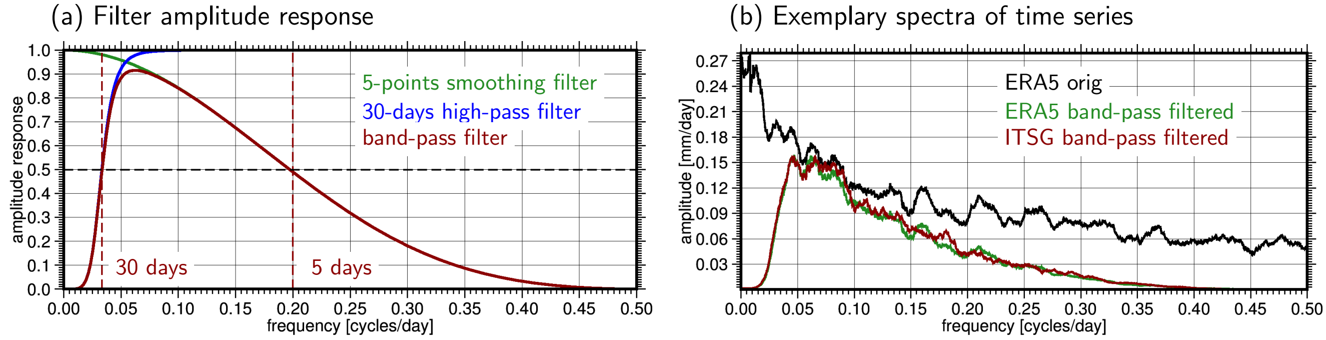

Figure 3. (a) Amplitude response of the low-pass filter (green), the high-pass filter (blue), and the resulting band-pass filter (red). (b) Spectra of the original ERA5 fluxes (black), the band-pass filtered ERA5, and the band-pass filtered ITSG-Grace2018 time series for an exemplary grid cell around the city of Aruanã, Brazil (S 15°, W 51°). A moving average (51 points) has been applied to all displayed spectra.

relationships between time series and thus can give valuable information about the agreement between GRACE and ERA5. However, it has the disadvantage of not being sensitive to differences in amplitude.

The root mean squared deviation (RMSD) provides a measure for the absolute differences between a signal x (here the reanalysis data) and a reference signal y (here the GRACE time series) according to

$$\mathrm{RMSD} = \sqrt{\frac{1}{I}\sum (x_i - y_i)^2}. \tag{3}$$

The RMSD is sensitive to differences in both amplitude and phase of the two analyzed time series. However, it should be noted that it depends on the magnitude of the signal and rather large RMSD values can be expected in areas with large flux variations, even though relative to the signal magnitude the agreement might be quite good. Therefore, as a third metric the explained signal variance VAR describes the ratio of the standard deviations of the differences between the two time series and the signal RMS. It can be interpreted as information about how much of the reference GRACE data can be explained by the reanalyses,

$$\mathrm{VAR} = 1 - \frac{\sum [(x_i - \bar{x}) - (y_i - \bar{y})]^2}{\sum (y_i - \bar{y})^2}. \tag{4}$$

The explained variance can have values of $-\infty < \mathrm{VAR} < 1$ with $\mathrm{VAR} = 1$ corresponding to a perfect match between the two time series. Since in our study each time series is centered around zero ($\bar{x} = \bar{y} = 0$) the explained variance equals the Nash-Sutcliffe efficiency often used for characterizing hydrological model performance. Values of $\mathrm{VAR} > 0$ mean that the reanalysis fits better to GRACE than a time series with a constant value of zero.

S3: Spatial Leakage at the Coasts

Results for all three evaluation metrics introduced above are displayed for the comparison of GRACE and ERA5 at all continental 1° grid cells (Fig. 4). While in large parts of the land areas the RMSD values (Fig. 4b) are in the range of 1.2 to 3 mm/day, the map is dominated by very large discrepancies between GRACE and ERA5 along the coast lines, especially in the Northern hemisphere at Alaska's west coast, Hudson Bay, North Sea and Baltic Sea in Europe, Black Sea, Persian Gulf, and large parts of the northern and eastern coasts of Asia. Similar features can be observed along the south-eastern coast of South America and in Northern Australia. These patterns can be assumed to be spurious high-frequency ocean signal being leaked onto land areas during the GRACE data processing, which is typically referred to as leakage-in in the geodetic literature[1]. The map of the explained variances (Fig. 4c) also reveals a second effect related to spatial leakage: In regions with very small oceanic variability, such as the equatorial Atlantic and the Indian Ocean, the GRACE signal is dampened along the coasts. This small variability of the reference signal results in a very small denominator in the fraction in Eq. (4) leading to strongly negative values of VAR. Particularly large negative values (the global minimum is -48.4) caused by this dampening effect can be found, e.g., around the equatorial coasts of Africa.

As the erroneous coastal zones hamper the computation of realistic global metrics, we limited our investigation area to continental regions not too strongly influenced by ocean leakage. We therefore performed the following two analysis steps: (1) We developed a land-ocean mask (grid values of one on the continents and zero on the oceans) into a spherical harmonic

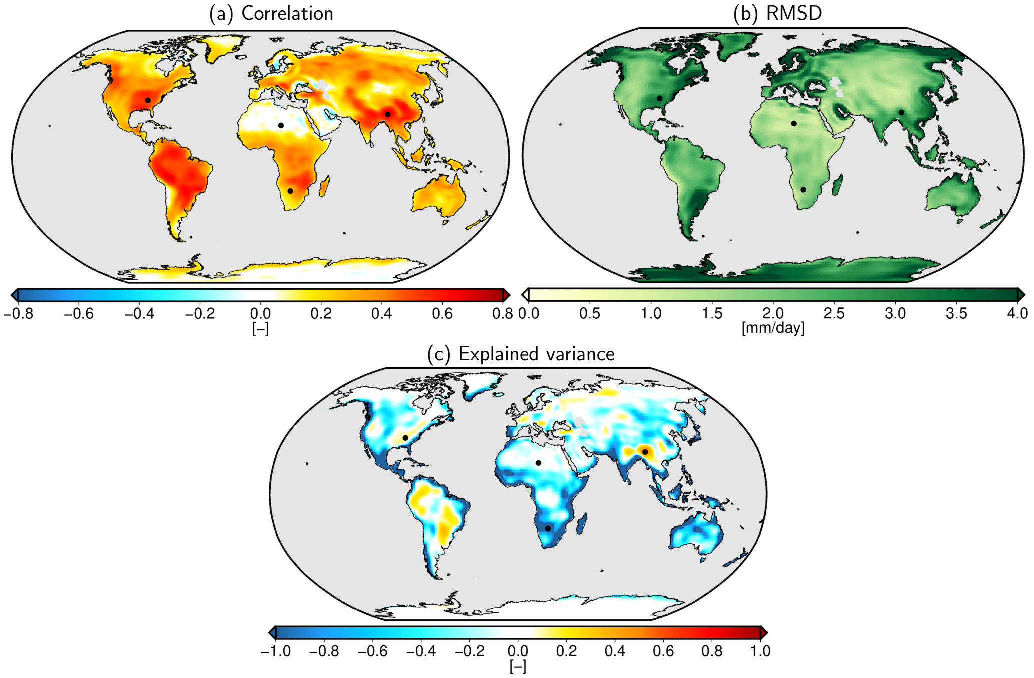

Figure 4. Validation metrics applied for comparing ERA5 with GRACE: (a) correlation coefficient with non-significant grid cells shaded, (b) RMSD, (c) explained variance. The positions at which the time series for ERA5 and GRACE are compared in Fig. 6 are shown as black dots in each of the figures.

expansion of degree $n = 40$, see Fig. 5a, and chose a threshold of 0.8 for masking out all coastal areas too strongly affected by leakage. (2) A second effect is caused by the Kalman smoother processing approach: In the process model used as the basis for the daily parameter estimation a specific signal covariance matrix is assumed to represent the expected signal characteristics both on oceans and continents. Due to the limited spectral resolution of the GRACE time series, there will be non-zero variances on land, even if the continental hydrology component of the process model is neglected. The ocean signal covariance matrix was propagated from storage to flux using the same 5-points differentiation filter applied in converting the GRACE water storage time series to water fluxes. The propagated ocean flux variances are shown in Fig. 5b and c, where the leakage effect onto the land areas can be observed. Especially in regions with strong signals, complex coastlines, and semi-enclosed seas, we find standard deviation values up to 15 mm/day on the continents. As no high-pass filter was applied in the variance propagation, the propagated flux variance is a conservative estimate as it also contains the temporal long wavelength information. Here a threshold of 3.5 mm/day was chosen to remove the most strongly affected grid cells (Fig. 5c), which in some regions slightly extends over the areas identified in step (1). The resulting mask in Fig. 5d shows in blue the areas that were excluded from further analysis due to the combination of both thresholds.

S4: Additional Flux Time Series

In addition to the flux time series computed for the exemplary grid point in Aruanã, Brazil (see Fig. 2 of the main text, Fig. 1 and 2 of the supplement), Fig. 6 shows the comparison of the band-pass filtered flux time series for ERA5 (green) and GRACE (red) in four additional geographical locations. These locations are indicated as black dots in Fig. 4 and were chosen to represent different climatic conditions and signal characteristics. The first point is located in the Brahmaputra River basin (N 27°,E95°, Fig. 6a), a monsoon-dominated area facing heavy rainfall during the summer, where a very good agreement between GRACE and ERA5 is observed. Generally, this location exhibits a high correlation ($\rho = 0.64$ for the time span 2003–2015) and one of the highest values for explained variance (VAR= 0.41, see Fig. 4). Another location was chosen close to the city of Nashville in the United States (N 36°, W 87°, Fig. 6b) where fast flux fluctuations are found in both the ERA5 and GRACE time series throughout the year. The correlation in this grid cell for the full time series is quite large ($\rho = 0.49$), while the explained variance is almost zero (0.01). In the Kalahari Desert (S 22°, E 22°, Fig. 6c) longer dry periods are interrupted by occasional strong rainfall events. These peaks also show up in the GRACE signal, albeit with a smaller magnitude. Nevertheless, the overall explained variance is largely negative (VAR= −1.50) in this grid cell. The last example is located in the Sahara Desert (N 20°, E 15°, Fig. 6d) where the very low signal strength prohibits a reasonable evaluation of reanalyses fluxes by GRACE.

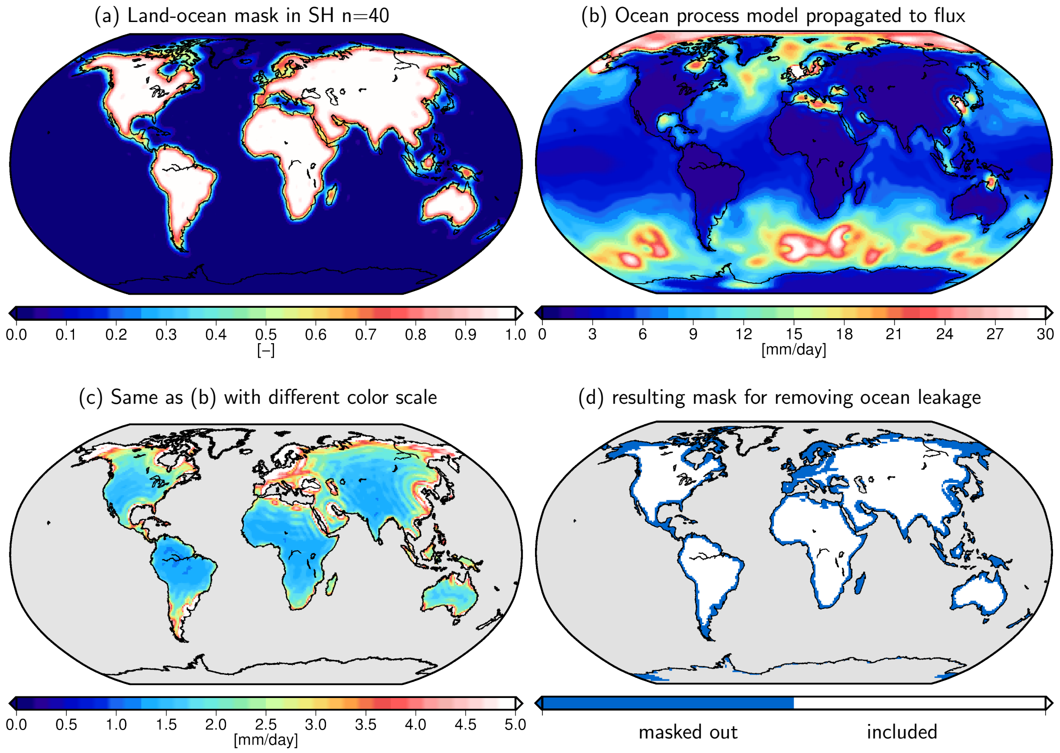

Figure 5. (a) Land/ocean mask of ones/zero developed into a spherical harmonic representation up to degree $n = 40$, (b) auto-covariance of ocean process model used in Kalman filtering to compute ITSG-Grace2018 daily solutions propagated from storage to flux, (c) same as (b) but with different color scale and restricted to continental area to show influence of ocean signal on land, (d) resulting mask to eliminate leakage affected areas.

S5: Investigate the influence of the GRACE signal-to-noise ratio

The explained variances (VAR) have shown to be largely negative when the full GRACE time series is taken into account (Fig. 3 c and 3f of the main text). The very different numbers obtained for individual seasons in the exemplary time series shown in Fig. 2 of the main text (i.e. positive VAR in the rainy season and negative VAR in the dry season) suggest that the results are strongly influenced by months of low GRACE signal variability and thus an unfavorable signal-to-noise ratio (SNR). To further investigate this issue, we repeated the computation of the VAR grids and excluded for each grid cell individual months, in which the GRACE signal variability RMS is smaller than a certain threshold (Fig. 7). The results show that while for the full times series only 19% of the continental area has an explained variance larger than zero with a maximum value of 0.45, the correspondence of reanalysis and satellite data increases substantially when raising the threshold. Maximum values of 0.59 are obtained for a threshold of 1.8 mm/day, and even 0.91 for a threshold of 2.6 mm/day. For large thresholds, some regions with very small signals (e.g. desert areas) get completely excluded from the analysis, as in individual grid cells the signal variability does not reach above the threshold for any month of the time series (greyed out in Fig. 7). Summarizing the results, Fig. 8 shows how the fraction of land area with positive VAR values increases with increasing threshold and how the fraction of land area taking part in the analysis decreases. From a minimum signal RMS of 1.8 mm/day upwards, the explained variance of the remaining grid cells becomes largely positive. The Sahara test performed in Section 2 of the paper revealed that this variance can be regarded as the upper bound of the noise range of GRACE. When the SNR becomes even larger, the fraction of positive VAR values increases towards 62% (threshold of 2.2 mm/day) or even 72% (threshold of 2.6 mm/day). In the latter case, however, the threshold is only reached in 68% of the land area. Independent of the choice of the threshold, the explained variance is substantially larger for ERA5 than it is for ERA-Interim, thereby fully confirming the conclusions outlined in the main text.

S6: Impact of Removing Atmospheric Mass Variations

Satellite gravimetry as realized with GRACE is principally unable to discriminate between mass anomalies above, at, or beyond the surface of the Earth. The gravitational effects of mass variability in the atmosphere are therefore removed by an a priori

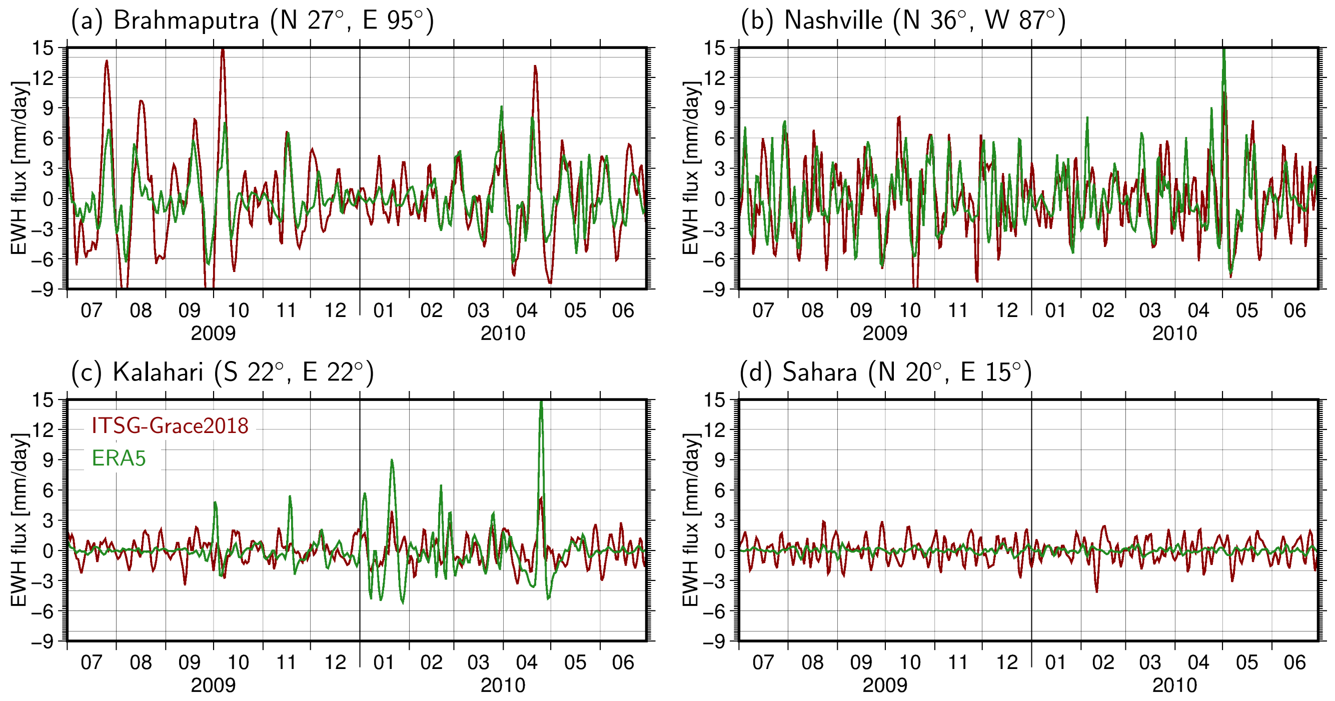

Figure 6. Comparison of the high-pass filtered flux time series derived from ERA5 (green) and GRACE (red) in four additional locations.

model from the sensor data before the inversion of the gravity field that is expected to contain only signals of variations in terrestrial water storage. For the a priori model AOD1B RL06 as applied in ITSG-Grace2018, 3-hourly data from ERA-Interim is used before Jan 1st, 2007, followed by operational ECMWF data afterward. Details on the processing of AOD1B are given in the product description document (`ftp://isdcftp.gfz-potsdam.de/grace/DOCUMENTS/Level-1/GRACE_AOD1B_Product_Description_Document_for_RL06.pdf`).

As outlined in Ch. 4 of the product description document, AOD1B considers the gravitational effect of upper-air density variations by vertically integrating over temperature and humidity data given at hybrid sigma level surfaces. The effect is very small when compared to the contribution of the surface pressure variations, amounting to just 4% of the overall signal even for extreme cases. For atmospheric surface pressure, we note that in situ pressure observations with high precision and dense geographical coverage are abundantly available for most continental areas. The comparatively high weights applied to those observations leads to the fact that surface pressure analyses from the ECWMF are closely aligned to other state-of-the-art global reanalyses as long as effects from different model orographies are properly accounted for[2,3]. We thus expect that the assessment of atmospheric fluxes with GRACE is not positively biased towards ERA-Interim despite of the fact that this re-analysis also contributed to the de-aliasing model. We moreover note that ERA5 information has not been made available when the current version of AOD1B was released, so that a positive bias towards ERA5 is hardly possible.

S7: Number of GRACE Observations per Day

Fig. 9 shows the number of available GRACE observations per day that were used in the daily Kalman smoother results. The frequent data gaps starting in 2011 were caused by instrument shutdowns due to waning battery capacities. On 727 days in the time span 2003—2015 (4744 days) less than 10,000 observations (about 18,000 per regular day) contributed to the solution. We exclude these 15% of days with insufficient observation count prior to the analysis.

S8: Complementary Land Surface Scheme Simulations

The water balance equation (Eq. 1) utilized in this study is known to be valid only at large spatial scales of river catchments. In order to confirm its validity for the particular spatial and temporal scales considered in this study, we perform two numerical experiments with the Land Surface and Discharge Model (LSDM[4]) with daily atmospheric forcing fields obtained from both ERA-Interim and ERA5. LSDM is based on numerical codes developed originally at the Max-Planck-Institute for Meteorology in Hamburg, Germany[5,6], which have been used already in the past to validate water fluxes from atmospheric re-analyses[7].

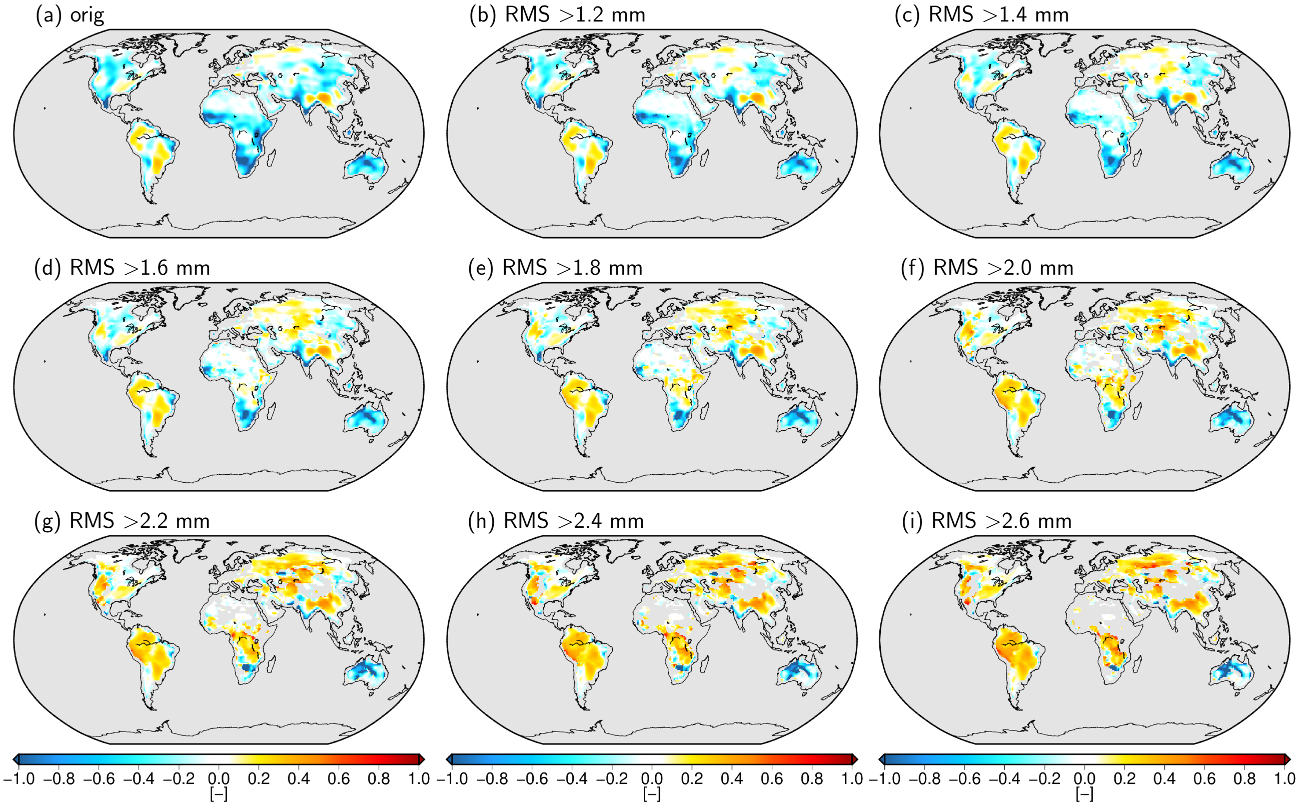

Figure 7. Explained variance of the GRACE signal by ERA5 when excluding months with GRACE signal variability below a certain threshold. The original result taking into account the complete time series is shown in the top left for comparison (same as Fig. 3f of the main text). The threshold is indicated by the RMS values given for each figure.

LSDM is in particular utilized for geodetic applications[8,9] in a global configuration discretized at a 0.5° regular grid. The model uses precipitation, evaporation and 2m-temperatures with daily temporal sampling as external forcing. Precipitation is disaggregated into rain and snow, thereby allowing to calculate snow accumulation, snowmelt, rainmelt, re-freezing, throughfall, and drainage. At places with open water surfaces, evaporation as given from the atmospheric forcing is increased up to the amount of potential evaporation given by Thornthwaite's equation using 2m-temperatures. Evaporation is further adjusted to the water amount available in the near-surface soil. Any residual water mass is stored as soil moisture, groundwater, or in rivers and lakes. The storage is subsequently discharged by a bucket-type runoff scheme through the river network towards the oceans. Glaciated regions are treated as permanently snow covered areas with a time-invariant ice-sheet below.

In order to demonstrate the suitability of the water balance equation for the validation of atmospheric water fluxes, we utilize simulated water storage variations from two LSDM experiments forced with ERA-Interim and ERA5. These storage variations were converted to fluxes by applying the same 5-points differentiation filter as used for the GRACE storages. The resulting apparent atmospheric fluxes from LSDM were further expanded into spherical harmonics up to degree and order 40, and subsequently high-pass filtered to isolate the sub-monthly time scales. The simulated fluxes are still dominated by ERA5 or ERA-Interim precipitation, but differ from the original reanalyses fluxes $P-E-R$, since they also include all apparent fluxes induced by the simulated surface water dynamics. A comparison of atmospheric fluxes from LSDM to GRACE is shown in Fig. 10. With respect to Fig. 3 of the main text, we note that all individual metrics for ERA5 and ERA-Interim (upper and middle row) are highly similar to the results obtained directly from the reanalyses. This nicely corroborates that the omission of lateral runoff (and all other simplifications contained in Eq. 1) indeed does not degrade our results. On the contrary, the improvements of ERA5 over ERA-Interim (bottom row) are even more pronounced when investigating the reanalyses fluxes directly. We thus conclude that satellite gravimetry can provide a direct assessment of water fluxes given by an atmospheric re-analysis without the need to introduce a – to some extent subjectively selected and potentially systematically biased – land surface scheme.

In addition, we note small differences between the validation results obtained from the simplified water balance equation

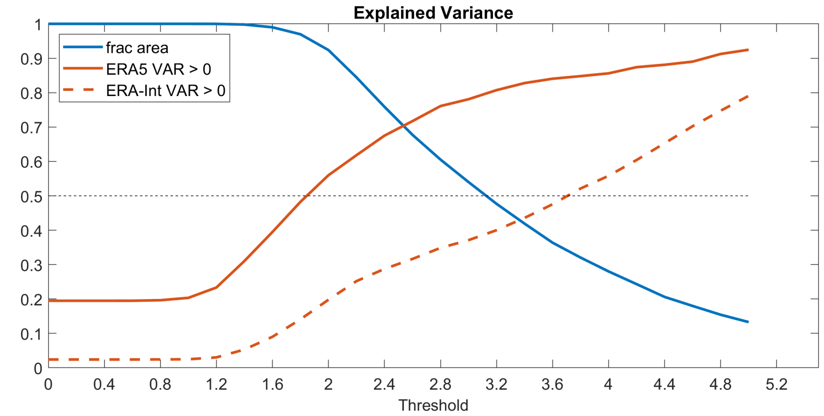

Figure 8. Fraction of land area with positive VAR values for ERA5 (solid red line) and ERA-Interim (dashed red line) in dependence of the chosen threshold for excluding months with GRACE small signal variability. The blue line indicates the fraction of the land area in which the signal variability reaches above the respective threshold.

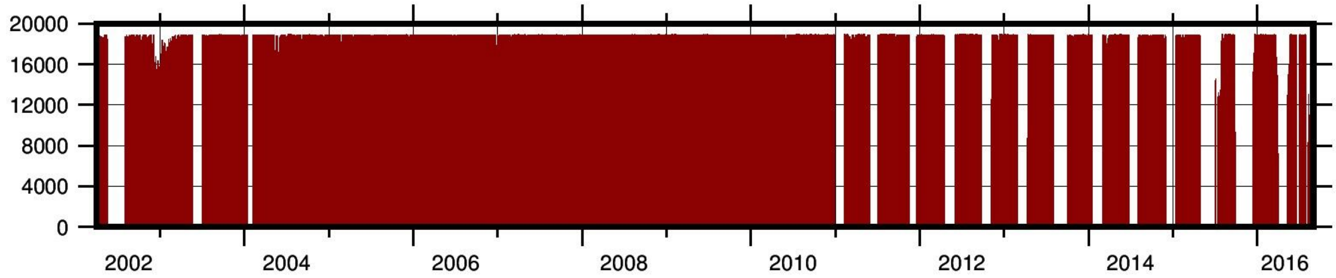

Figure 9. Number of GRACE observations per day

and LSDM in particular at mid latitudes as, e.g., South Africa. We recall that precipitation is a prescribed forcing data-set to LSDM, whereas evaporation from the forcing is modified by the model in order to better account for the water availability at the surface in particular in regions with open water surfaces. It is beyond the scope of this study to analyze in detail the spatial and temporal variability of the evaporation increments simulated by LSDM obtained from the two experiments, but we nevertheless conclude that the utilization of a land surface scheme – or even a small model ensemble as utilized by [10] – would provide an opportunity to validate even the atmospheric water flux components precipitation and evaporation individually with satellite gravimetry data.

S9: Evaluation of previous GRACE solutions

In Fig. 3 of the main paper we showed that ITSG-Grace2018 can distinctively detect quality differences between ERA5 and its predecessor model ERA-Interim. We repeated the whole analysis with an older release of the daily GRACE time series (ITSG-Grace2016[11]). The results (Fig. 11) reveal that a quality assessment of high-frequency atmospheric fluxes had not yet been possible with previous GRACE releases. Firstly, the comparison of ITSG-Grace2016 with both reanalyses results in smaller correlations and larger RMSD values than it was the case for ITSG-Grace2018 (upper two rows of Fig. 11 compared to Fig. 3 of the paper). Secondly, the differences between ERA5 and ERA-Interim (bottom row) do not provide a clear picture: The correlation differences (Fig. 11g) show a presumably random pattern of positive and negative values. The extent of areas in the RMSD difference map (Fig. 11h) that reveal degradation (yellow) is almost as large as those areas indicating improvement (blue). The differences in explained variance (Fig. 11i) are generally positive, but in much smaller parts of the continental areas than this is the case for the 2018 GRACE solution (Fig. 3i of the paper). We thus conclude that only the recent progress in GRACE data processing has enabled the use of daily GRACE time series for evaluating high-frequency atmospheric fluxes.

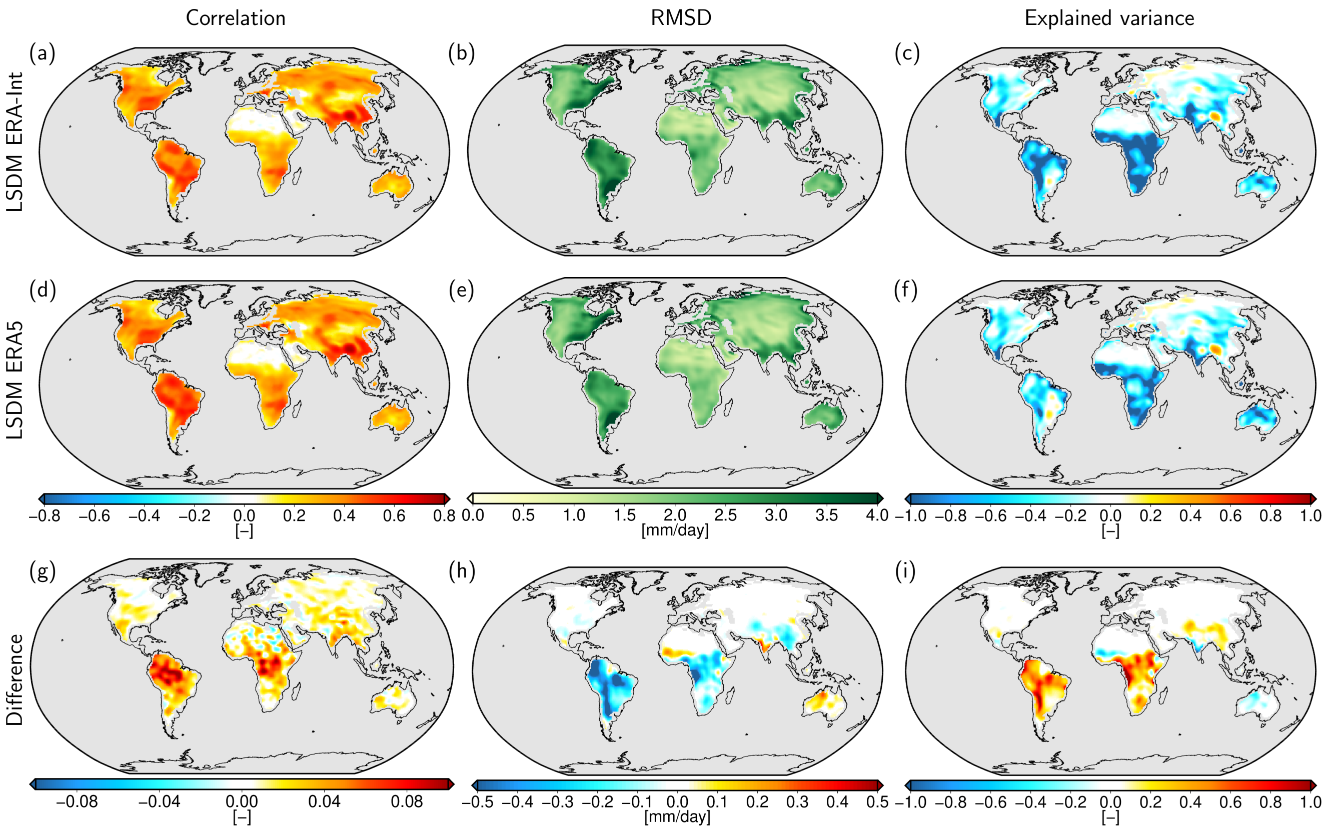

Figure 10. Comparison of three evaluation metrics between GRACE and apparent fluxes derived from LSDM simulations with ERA-Interim and ERA5 atmospheric forcing: correlation coefficient (left column), root mean squared deviation (middle column), and relative explained variance (right column). The top row shows the respective error measures for ERA-Interim forcing, the middle row for ERA5 forcing and the lower row the difference for each metric (ERA5 minus ERA-Interim forcing). Coastal regions potentially affected by ocean leakage are masked out.

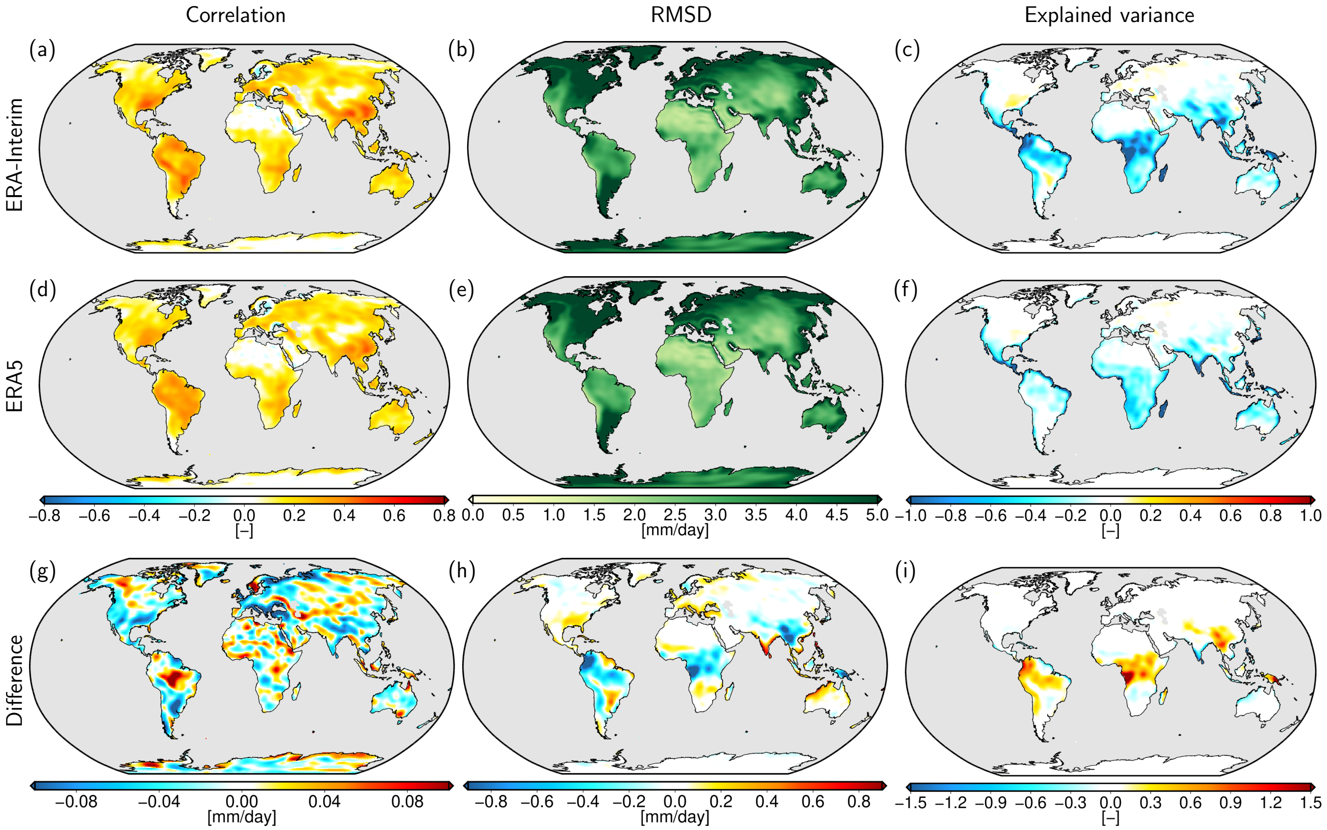

Figure 11. Comparison of three evaluation metrics between reanalyses and ITSG-Grace2016: correlation coefficient (left column), root mean square deviation (middle column), and relative explained variance (right column). The top row shows the respective error measures for ERA-Interim, the middle row for ERA5 and the lower row the difference for each metric (ERA5 minus ERA-Interim).

References

1. Baur, O., Kuhn, M. & Featherstone, W. E. GRACE-derived ice-mass variations over Greenland by accounting for leakage effects. *J. Geophys. Res. Solid Earth* **114**, DOI: 10.1029/2008JB006239 (2009).
2. Dobslaw, H. *et al.* Modeling of present-day atmosphere and ocean non-tidal de-aliasing errors for future gravity mission simulations. *J. Geod.* **90**, 423–436, DOI: 10.1007/s00190-015-0884-3 (2016).
3. Dobslaw, H. Homogenizing surface pressure time-series from operational numerical weather prediction models for geodetic applications. *J. Geod. Sci.* **6**, DOI: 10.1515/jogs-2016-0004 (2016).
4. Dill, R. Hydrological model lsdm for operational earth rotation and gravity field variations. In *Scientific Technical Report STR08/09* (Deutsches GeoForschungsZentrum GFZ. Potsdam, 2008).
5. Hagemann, S. & Dümenil, L. A parametrization of the lateral waterflow for the global scale. *Clim. Dyn.* **14**, 17–31, DOI: 10.1007/s003820050205 (1997).
6. Hagemann, S. & Gates, L. D. Improving a subgrid runoff parameterization scheme for climate models by the use of high resolution data derived from satellite observations. *Clim. Dyn.* **21**, 349–359, DOI: 10.1007/s00382-003-0349-x (2003).
7. Hagemann, S. & Gates, L. D. Validation of the hydrological cycle of ECMWF and NCEP reanalyses using the MPI hydrological discharge model. *J. Geophys. Res. Atmospheres* **106**, 1503–1510, DOI: 10.1029/2000JD900568 (2001).
8. Dill, R., Dobslaw, H. & Thomas, M. Combination of modeled short-term angular momentum function forecasts from atmosphere, ocean, and hydrology with 90-day EOP predictions. *J. Geod.* **87**, 567–577, DOI: 10.1007/s00190-013-0631-6 (2013).
9. Dill, R., Klemann, V. & Dobslaw, H. Relocation of River Storage From Global Hydrological Models to Georeferenced River Channels for Improved Load-Induced Surface Displacements. *J. Geophys. Res. Solid Earth* **123**, 7151–7164, DOI: 10.1029/2018JB016141 (2018).
10. Zhang, L. *et al.* Validation of terrestrial water storage variations as simulated by different global numerical models with GRACE satellite observations. *Hydrol. Earth Syst. Sci.* **21**, 821–837, DOI: https://doi.org/10.5194/hess-21-821-2017 (2017).
11. Mayer-Gürr, T. *et al.* ITSG-Grace2016 - Monthly and Daily Gravity Field Solutions from GRACE, DOI: 10.5880/icgem.2016.007 (2016).
